# Effect of intranasal insulin on postoperative delirium: A systematic review and dose–response meta-analysis

**DOI:** 10.1016/j.ccrj.2026.100227

**Published:** 2026-10-27

**Authors:** Omar Saeed, Mohamedhen Vall Nounou, Abdelrahman Saeed, Bram Rochwerg, Ali M. Abdelaziz, Gerald L. Weinhouse, Sung Ryul Shim, Muhammed Elhadi

**Affiliations:** a Faculty of Medicine, Al-Azhar University, Damietta, Egypt; b Faculty of Medicine, University of Nouakchott Al Aasrya, Nouakchott, Mauritania; c Department of Medicine, McMaster University, Hamilton, Ontario, Canada; d Department of Health Research Methods, Evidence and Impact, McMaster University, Hamilton, Ontario, Canada; e Knowledge Centre, Hamilton Health Sciences, Hamilton, Ontario, Canada; f Faculty of Medicine, Alexandria University, Alexandria, Egypt; g Department of Medicine, Brigham and Women's Hospital, Boston, MA, USA; h Department of Biomedical Informatics, Konyang University College of Medicine, Daejeon, South Korea; i College of Medicine, Korea University, Anam-ro, Seongbuk-gu, Seoul, 02841, South Korea

**Keywords:** Intranasal insulin, Postoperative delirium, Dose–response meta-analysis, Surgery, Randomised controlled trial

## Abstract

**Objective:**

Postoperative delirium (POD) is a common neurological complication associated with adverse clinical outcomes. Intranasal insulin may improve cognitive function, but its effectiveness and optimal dose for preventing POD remain uncertain. We evaluated the effect of intranasal insulin on POD and explored its dose–response relationship.

**Review methods:**

PubMed, Scopus, Web of Science, and the Cochrane Library were searched through November 2025. Randomised controlled trials comparing intranasal insulin with placebo in adult surgical patients were included. Random-effect pairwise and one-stage dose–response meta-analyses were performed. Risk of bias was assessed using the Cochrane Risk of Bias 2 tool, and certainty of evidence was evaluated using the Grading of Recommendations Assessment, Development, and Evaluation framework.

**Results:**

Eight randomised controlled trials involving 892 patients were included (502 receiving intranasal insulin and 390 receiving placebo). Intranasal insulin probably reduces POD (moderate-certainty evidence) compared with placebo (risk ratio, 0.39; 95% confidence interval, 0.29–0.51). Subgroup analyses found no significant differences according to surgery type or anaesthetic technique. A dose–response analysis demonstrated a nonlinear association, with a rapid reduction in POD risk at lower doses and the greatest modelled benefit at 30 IU (risk ratio, 0.39; 95% confidence interval, 0.29–0.53). The dose–response curve plateaued above 30 IU, with no additional reduction in POD risk at higher doses. Seven trials had a low risk of bias, while one raised some concerns because of bias in measurement of the outcome.

**Conclusions:**

Intranasal insulin probably reduces POD in adult surgical patients. The dose–response findings suggest that 30 IU may provide the greatest benefit, with no apparent additional reduction at higher doses. Larger multicentre trials are needed to confirm the optimal regimen and comprehensively evaluate safety.

## Highlights

• Eight randomised trials involving 891 surgical patients were analysed.
• Intranasal insulin reduced postoperative delirium versus placebo.
• Moderate-certainty evidence supported an estimated 61% risk reduction.
• The greatest modelled benefit occurred at an intranasal dose of 30 International Units (IU).
• Doses above 30 IU provided no additional reduction in delirium risk.

## 1 Introduction

Postoperative delirium (POD) is an acute, transient neuropsychiatric condition that develops within hours to days after surgery. Its diagnosis, per Diagnostic and Statistical Manual of Mental Disorders, Fifth Edition (DSM-5) criteria, requires a disturbance in attention and awareness that develops over a short period and represents a change from baseline; a fluctuating course; an additional disturbance in cognition; and evidence that the disturbance is a direct physiological consequence of surgery, anaesthesia, or another medical condition and is not better explained by a pre-existing or evolving neurocognitive disorder.^1^ It affects 15–53% of elderly surgical patients.^2^ Clinically, delirium represents a temporary dysfunction of brain activity characterised by fluctuating disturbances in attention, awareness, and cognition. Although affected patients can receive sensory information, impaired integration leads to inappropriate behavioural responses to their environment.^3^

Previous studies have identified multiple risk factors for POD, including sex, advanced age, body mass index (BMI), smoking, prior episodes of delirium, comorbid conditions, polypharmacy, and elevated C-reactive protein levels.^4^

Neuroinflammation is considered a key pathophysiological mechanism underlying POD. Surgical trauma triggers a systemic inflammatory response that may persist into the postoperative period, contributing to neurocognitive dysfunction. Alterations in neurotransmitter systems also appear to play a role, particularly involving acetylcholine, which is essential for memory, attention, and several interconnected brain circuits.^5^ In this context, intranasal insulin therapy has attracted attention because it has been shown to affect inflammatory and immune markers, suggesting activation of compensatory immune response mechanisms with potential clinical benefits.^6^

The brain is a major target of insulin signalling as adaptor molecules and insulin receptors are widely distributed throughout the central nervous system (CNS).^7^^,^^8^ Because insulin administration can modify brain function, it is evident that insulin exerts direct effects on the CNS.^8^ Animal studies using gamma counting and high-resolution phosphor imaging have demonstrated that intranasally administered insulin-like growth factor-1 rapidly reached multiple regions of the brain and spinal cord.^9^

Further evidence indicates that intranasal insulin can bypass the blood–brain barrier and reach the CNS within approximately 1 h of administration.^10^ Following intranasal instillation, insulin is transported directly to the CNS along perivascular and perineural channels associated with the olfactory (cranial nerve I) and trigeminal (cranial nerve V) nerves, crossing the cribriform plate and entering the perivascular spaces of the olfactory bulb and brainstem. This pathway bypasses both the blood–brain barrier and hepatic first-pass metabolism, allowing insulin to appear in the cerebrospinal fluid within approximately 30–60 min of administration, without clinically meaningful increases in systemic insulin concentration.^10^ Unlike systemic insulin, which reaches the CNS through limited, saturable blood-to-brain transport, intranasal insulin can provide rapid CNS exposure through direct nose-to-brain pathways. In healthy adults, intranasal insulin increased cerebrospinal fluid insulin concentrations within minutes without significantly increasing serum insulin or lowering plasma glucose, supporting the possibility of central delivery with limited peripheral metabolic effects.^10^ This delivery route has become increasingly common in both preclinical and clinical research, offering a safe, noninvasive method for targeting the brain.[11], [12], [13] Eight weeks of intranasal administration of regular insulin has been shown to improve memory in human studies.^14^ As a relatively novel therapeutic approach, intranasal insulin has been proposed for the management of brain injury, cerebral ischaemia, and potentially POD and its associated adverse outcomes.^15^

The European Society of Anaesthesiology and Intensive Care has recommended several pharmacological strategies for managing POD.^16^ However, intranasal insulin is not currently endorsed for POD prevention, largely due to limited and inconclusive evidence. Previous meta-analyses by Zhang et al.^17^ and Amin et al.^18^ have evaluated intranasal insulin for POD prevention.

Therefore, the aim of this systematic review and dose–response meta-analysis was to evaluate the effect of intranasal insulin on POD in adult surgical patients, with particular attention to dose–response relationships.

## 2 Methods

This systematic review and meta-analysis was conducted in accordance with the Preferred Reporting Items for Systematic Reviews and Meta-Analyses statement.^19^ We followed the Cochrane Handbook for Systematic Reviews of Interventions when conducting this review.^20^ Our Preferred Reporting Items for Systematic Reviews and Meta-Analyses checklist is shown in (Supplementary Table 1). We registered this systematic review and meta-analysis on the International Prospective Register of Systematic Reviews (CRD420250634326).

### 2.1 Search strategy

PubMed, Scopus, Web of Science, and the Cochrane Library were searched through November 2025. We included search keywords for intranasal insulin and POD. The full search strategy is mentioned in (Supplementary Table 2).

### 2.2 Study selection

Two independent authors initially conducted a title and abstract screening of the literature results and removed any duplicates using Covidence (Veritas Health Innovation, Melbourne, Australia). Afterwards, we performed a full-text screening using an Excel sheet. The opinion of a senior author resolved any disagreement between the authors.

### 2.3 Inclusion and exclusion criteria

We included any randomised clinical trial assessing the effect of intranasal insulin at any dose on POD in adult surgical patients (cardiac, orthopaedic, gastrointestinal) compared to placebo (saline). We excluded cohort, case–control, case series, case reports, reviews, animal studies, abstracts, editorials, commentaries, posters, and non-English studies.

### 2.4 Data extraction and outcomes

Two independent authors extracted relevant data into an extraction sheet. This sheet consisted of two parts: the first was a summary of the included studies, and the second included characteristics such as study ID, study design, country, sample size, insulin dose, type of surgery, age, sex, BMI, American Society of Anesthesiologists (ASA) classification, and comorbidities.

Our outcome was POD, assessed and defined using the Confusion Assessment Method (CAM) tools (CAM-ICU, 3D-CAM, 3D-CAM-CN). Any disagreement between the authors was resolved by discussion first; if a conflict persisted, the senior author’s opinion determined the outcome.

### 2.5 Risk of bias assessment

Two independent authors assessed the quality of the included studies using the Revised Cochrane Risk of Bias Tool (RoB 2) for randomised controlled trials (RCTs).^21^ The RoB 2 tool categorised each RCT as high, low, or some concerns according to the following five domains: (i) bias arising from the randomisation process; (ii) bias due to deviations from intended interventions; (iii) bias arising due to missing outcome data; (iv) bias in the measurement of the outcome; and (v) bias in the selection of the reported result. The opinion of a senior author resolved any disagreement between the authors.

### 2.6 Statistical analysis

We used R statistical software v4.0.3 to analyse the data. The R package meta v4.12-0 was used to perform pairwise meta-analysis. We used a random-effect model to account for variability across the included studies. Continuous data were expressed as mean differences when available, and dichotomous data as risk ratios (RRs), both with 95% confidence intervals (CIs). We assessed heterogeneity visually and used the chi-squared (χ^2^) test and I^2^ statistics to assess heterogeneity across the included studies. Using the R package “dosresmeta”, developed by Crippa and Orsini, we conducted a one-stage dose–response meta-analysis in a frequentist framework based on a restricted maximum likelihood random-effect model. We used restricted cubic splines with three knots at the 10th, 50th, and 90th percentiles of the distribution.^22^ We examined the association between POD and intranasal insulin dosage. The variance partition coefficient (VPC), a multivariate extension of the I^2^ value proposed by Crippa et al., was used to quantify heterogeneity in the dose–response meta-analysis. In addition to the primary pairwise and dose–response analyses of POD, we conducted subgroup dose–response analyses stratified by surgery type and anaesthetic technique, given the observed clinical heterogeneity in the surgical population, and these should be interpreted accordingly.

To assess the influence of variables associated with POD, we performed a multivariable one-stage dose–response meta-analysis of insulin dose and POD risk, adjusting for study-level covariates. Only variables with complete data were included in the analysis (age, sex, and BMI) as restricting adjustment to fully reported covariates avoids the bias and reduced precision associated with missing data.

### 2.7 Certainty of evidence

The Grading of Recommendations Assessment, Development, and Evaluation (GRADE) framework^23^ was used to appraise the quality of evidence, which was classified as very low, low, moderate, or high. We used the GRADE narrative language summary to contextualise results.^24^

### 2.8 Ethical approval

Not applicable. This study is a systematic review and meta-analysis based exclusively on data extracted from previously published RCTs. No individual patient data were collected, and the authors conducted no new human or animal studies.

## 3 Results

We identified 876 articles through a literature search across four databases: PubMed, Scopus, Web of Science, and Cochrane. Then, 344 duplicates were removed, and 532 articles were screened by title and abstract. Subsequently, 476 articles were excluded, leaving only 56 full-text articles for assessment according to predetermined inclusion criteria. Finally, eight trials[25], [26], [27], [28], [29], [30], [31], [32] were included in the meta-analysis (Fig. 1).

**Fig. 1:**
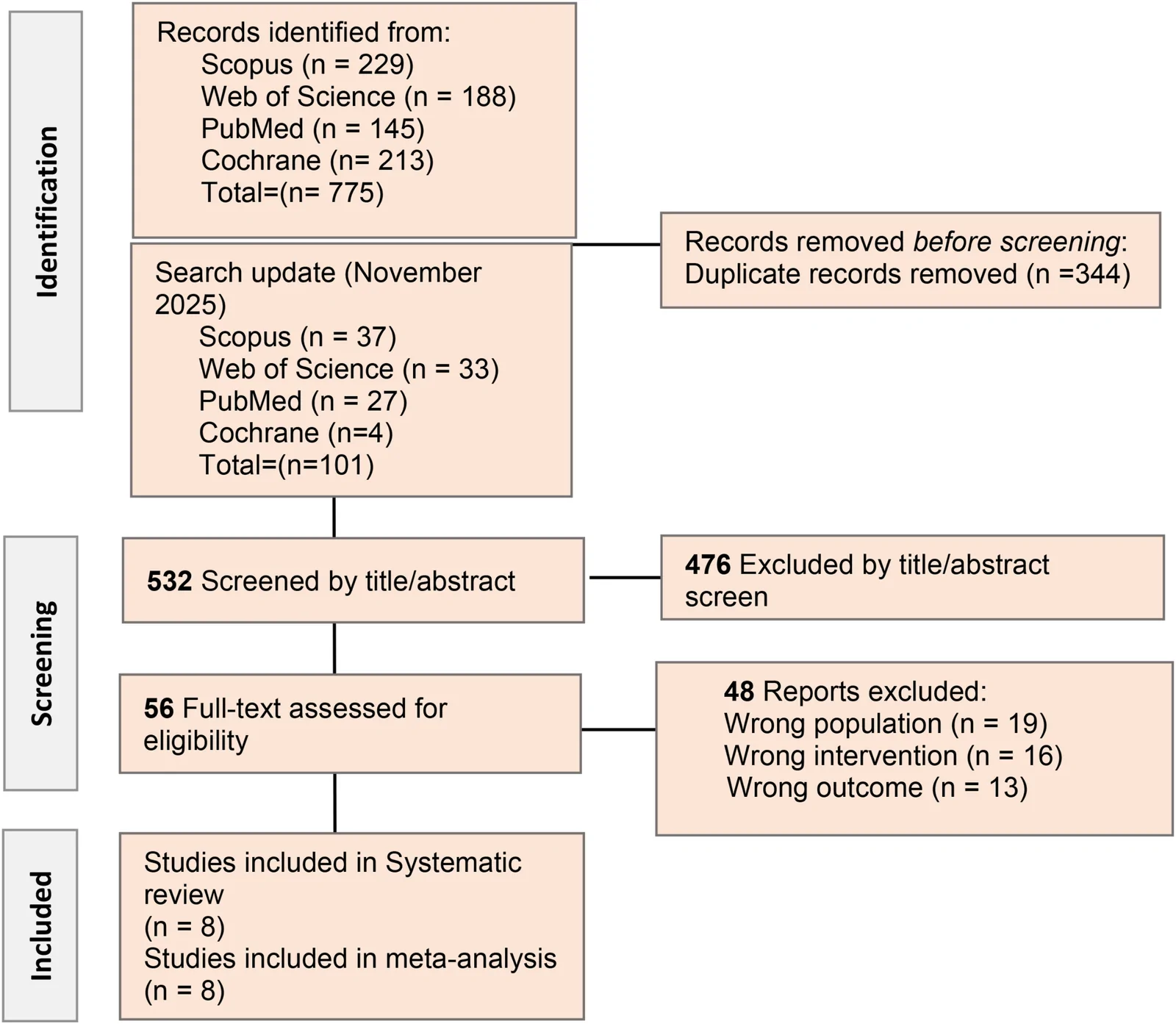
PRISMA flow diagram. PRISMA: Preferred Reporting Items for Systematic Reviews and Meta-Analyses.

### 3.1 Characteristics of included studies

This systematic review and meta-analysis included eight RCTs with a total of 892 patients; individual study sample sizes ranged from 71 to 195. All studies were conducted in China, except one in Canada.

The studies assessed the effect of intranasal insulin on POD compared with a control (saline), with doses ranging from 20 to 80 IU. Included surgeries in these studies varied from laparoscopic gastrointestinal tumour surgeries to cardiopulmonary bypass surgeries and joint replacement surgeries. Participant demographics showed a mean age range of 53–81.25 years across studies. The mean BMI ranged from 20.7 to 32.0 kg/m^2^, indicating a patient population comprising both normal-weight and obese individuals. The ASA physical status classification was reported in several studies, with most patients categorised as ASA II (moderate systemic disease). A comprehensive summary table (Supplementary Table 3) and baseline characteristics of the included studies (Supplementary Table 4) provide more details.

The dosing regimens of intranasal insulin differed significantly between the included studies; the timing and the number of doses are detailed in Supplementary Table 5.

### 3.2 Risk of bias assessment

We used the Cochrane RoB 2 tool to assess the quality of the eight included studies. Seven studies demonstrated low risk of bias across all five assessed domains, indicating robust methodology, while one study raised concerns due to bias in measurement of the outcome (Fig. 2).

**Fig. 2:**
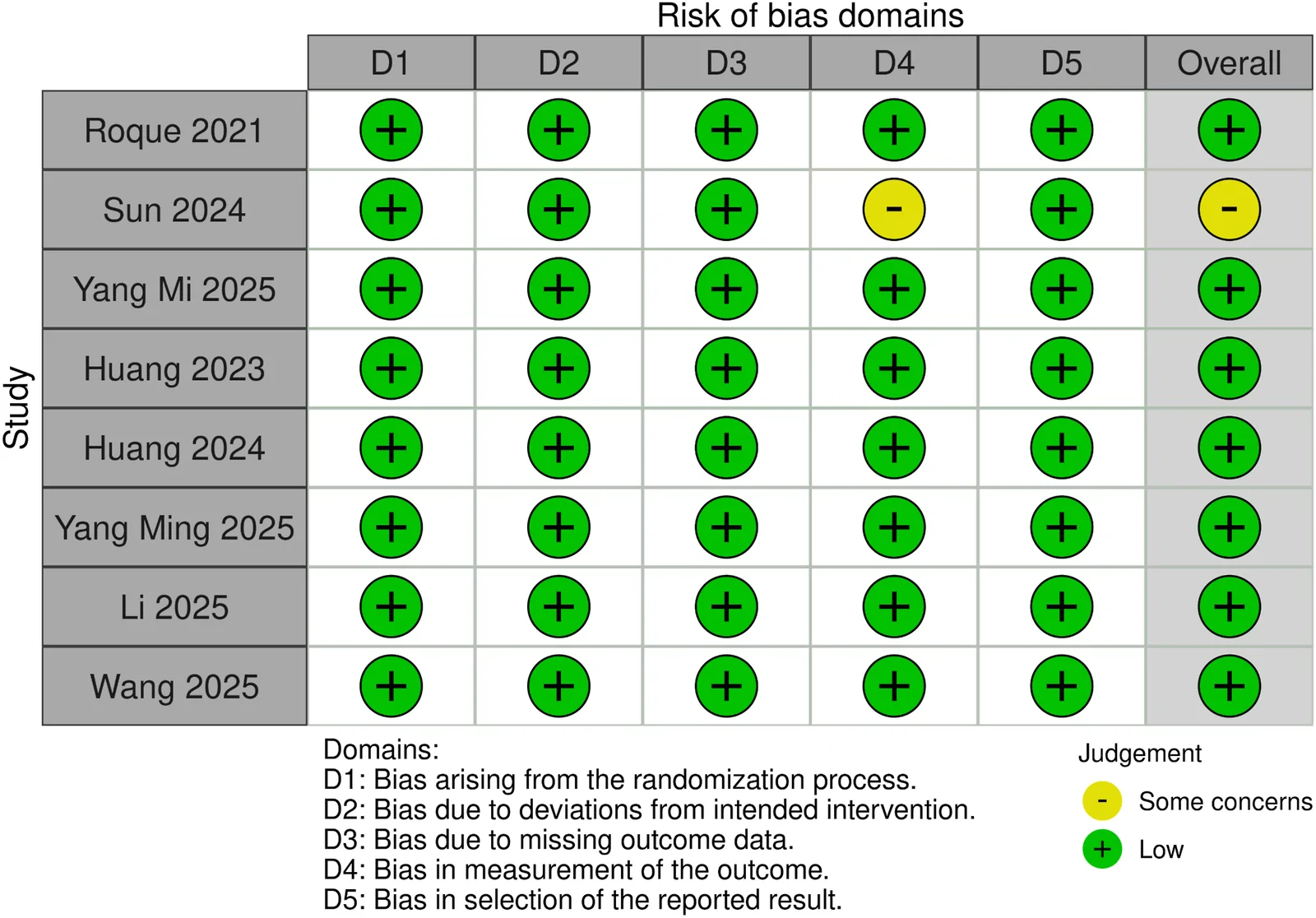
Risk of bias assessment.

### 3.3 Pairwise meta-analysis

The meta-analysis included eight trials with 891 patients (1 patient was not included in this analysis)(insulin: 501 vs placebo: 390) and showed that intranasal insulin probably reduces POD (moderate-certainty evidence) compared to placebo (RR: 0.39, 95% CI: 0.29 to 0.51) (Fig. 3). Subgroup analyses also revealed statistically significant differences across both surgery types (Supplementary Fig. 1) and anaesthetic techniques (Supplementary Fig. 2).

**Fig. 3:**
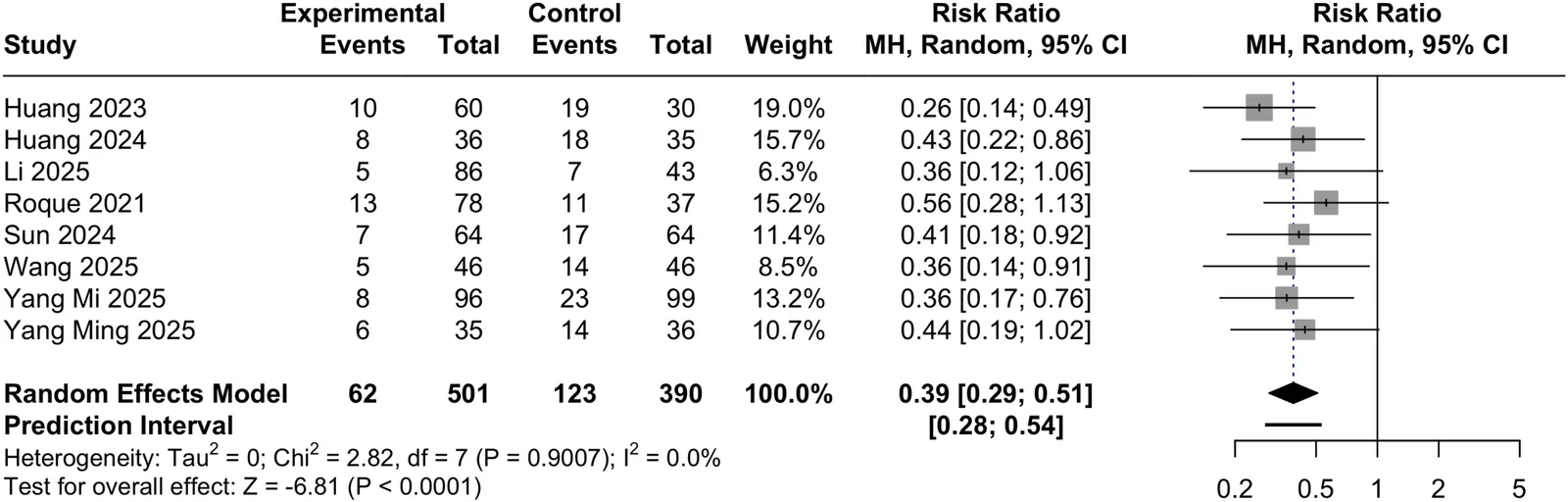
POD (pairwise meta-analysis). CI: confidence interval; POD: postoperative delirium; MH: Mantel-Haenszel.

### 3.4 Dose–response meta-analysis

This analysis included eight studies (dose range: 0–80 IU) and showed a nonlinear association between insulin dosage and POD. The curve showed a rapid reduction in POD risk with lower insulin doses, reaching the nadir at 30 IU (RR: 0.39, 95% CI: 0.29 to 0.53). However, the curve plateaued, and increasing the dose beyond 30 IU was not associated with a lower risk of POD (Fig. 4). The VPC curve showed no heterogeneity across the whole dose range (Fig. 5). A subgroup analysis by surgery type showed that the nadir for cardiac surgery was 29 IU (RR: 0.44, 95% CI: 0.27 to 0.72), and for orthopaedic surgery, the nadir was 22 IU (RR: 0.28, 95% CI: 0.11 to 0.70) (Fig. 6A). The subgroup analysis based on the type of anaesthesia revealed that the nadir for the general anaesthesia subgroup was 27 IU (RR: 0.40, 95% CI: 0.29 to 0.57), and the nadir for the regional anaesthesia was 20 IU (RR: 0.12, 95% CI: 0.02 to 0.92) (Fig. 6B). The linear and quadratic models for the dose–response meta-analysis are shown in Supplementary Figs. 3 and 4, respectively.

**Fig. 4:**
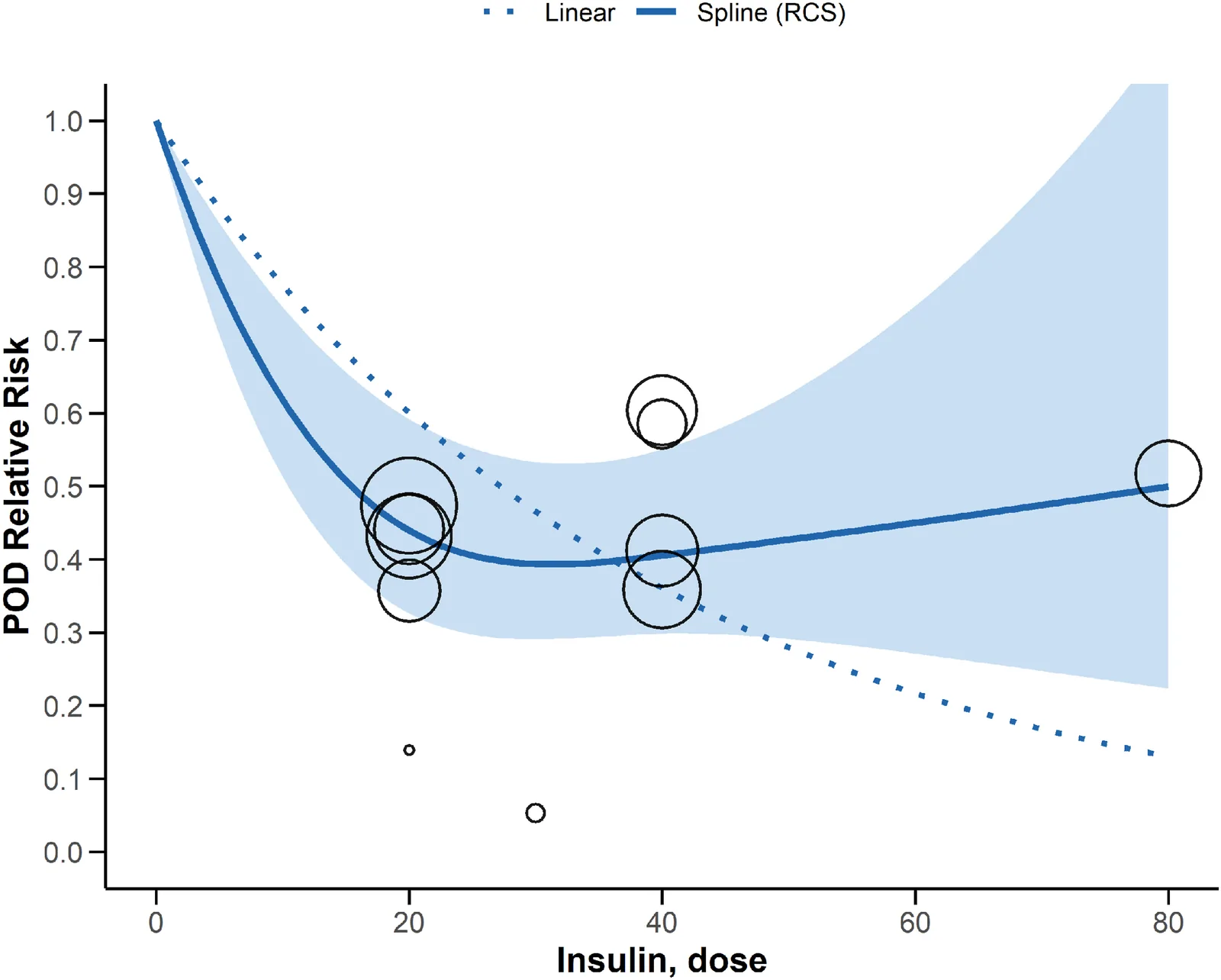
POD (Dose–response). POD: postoperative delirium; RCS: Restricted Cubic Spline.

**Fig. 5:**
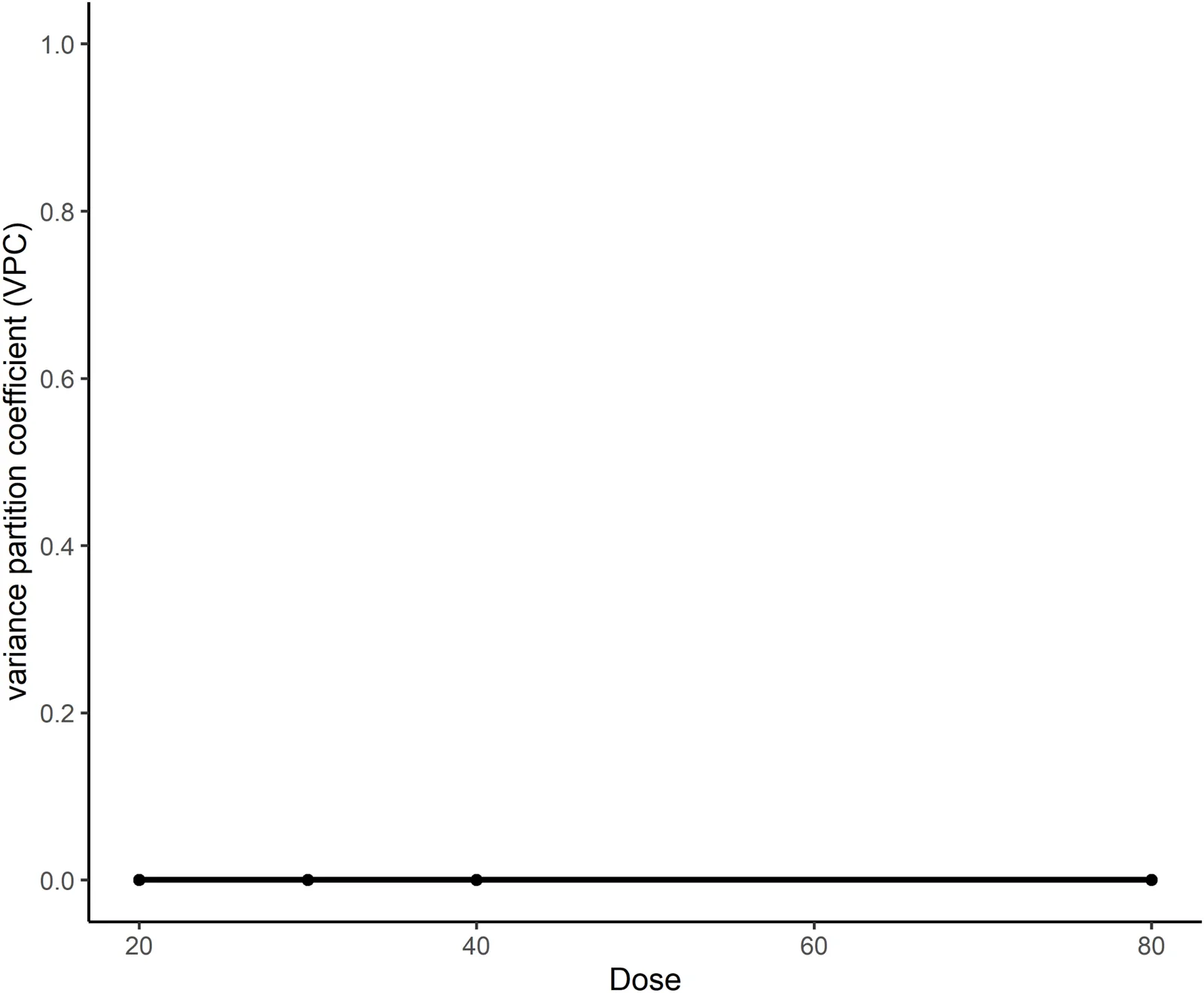
VPC curve. VPC: variance partition coefficient

**Fig. 6:**
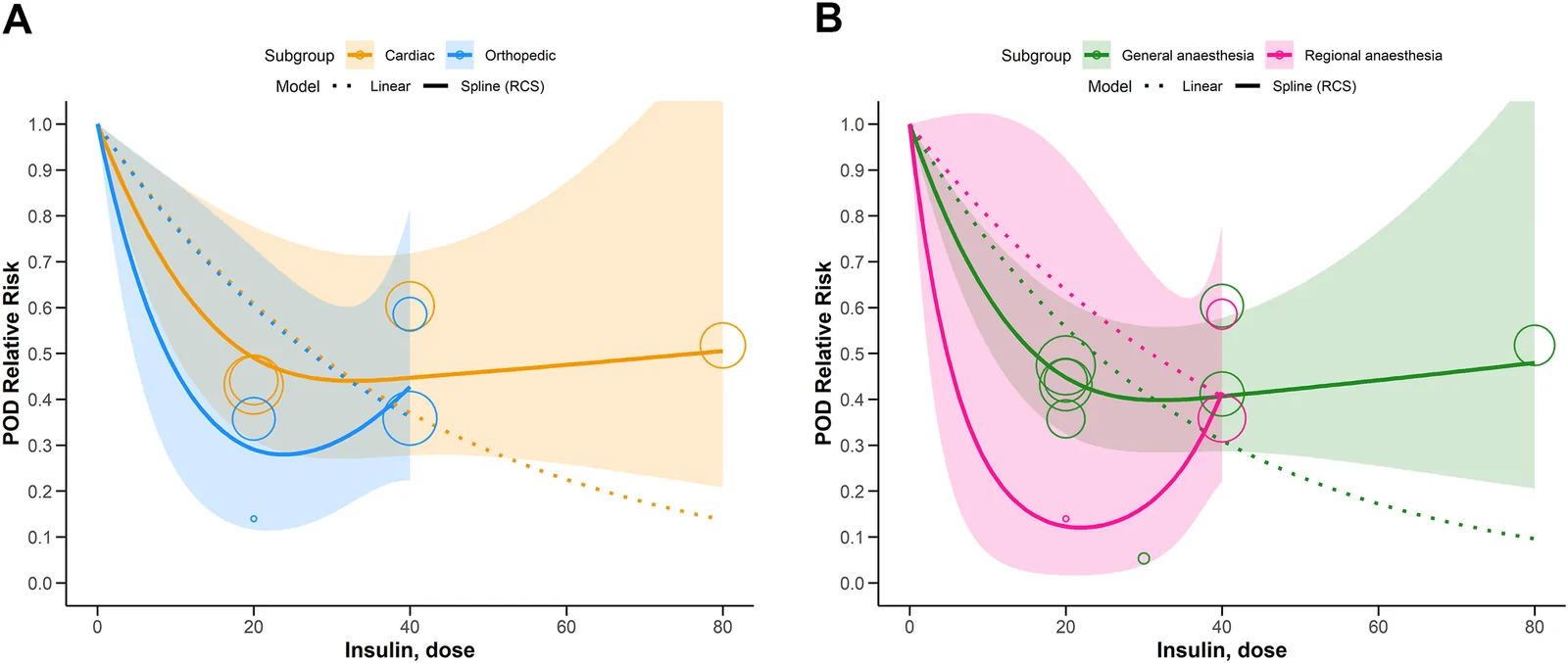
(A) Subgroups stratified by surgery type; (B) Subgroups stratified by anesthesia type. POD: postoperative delirium; RCS: Restricted Cubic Spline.

### 3.5 Certainty of evidence

The GRADE assessment showed that the evidence for POD had moderate certainty due to imprecision (Supplementary Table 6).

### 3.6 Multivariable analysis

We did not adjust for all baseline variables reported in the included studies because most of them (ASA grade, smoking status, alcohol use, left ventricular ejection fraction (LVEF), haematocrit, chronic obstructive pulmonary disease (COPD), atrial fibrillation, and diabetes mellitus) were missing or inconsistently reported and were available for only a limited number of the included studies. Age, sex, and BMI were the only baseline covariates reported by all eight included trials and were therefore the only variables entered as covariates in a multivariable one-stage dose–response meta-analysis.

After adjustment for study-level mean age, proportion male, and mean BMI, the overall shape of the dose–response curve was essentially unchanged from the unadjusted analysis. POD risk decreased steeply at lower insulin doses, and the adjusted nadir remained at 30 IU (adjusted RR: 0.38, 95% CI: 0.26 to 0.55), closely matching the unadjusted estimate (RR: 0.39, 95% CI: 0.29 to 0.53) (Supplementary Fig. 5). The adjusted VPC curve showed a modest, nonsystematic increase in residual heterogeneity at the lowest doses that was not present in the unadjusted model. However, heterogeneity across most of the dose range remained low (Supplementary Fig. 6).

Because only age, sex, and BMI could be incorporated, this multivariable analysis should be interpreted as a partial adjustment rather than a full adjustment for the confounders. ASA grade, smoking, alcohol use, and the other factors listed in Supplementary Table 4 could not be adjusted for in this study-level meta-analysis because they were not uniformly reported across the included trials.

## 4 Discussion

The aim of this systematic review and dose–response meta-analysis was to explore the effect of intranasal insulin and, explicitly, the impact of different doses on POD. We found that intranasal insulin, compared with placebo, probably reduces POD (moderate-certainty evidence) in adult surgical patients, regardless of insulin dose. Additionally, the dose–response meta-analysis showed that the optimal dose associated with the lowest incidence of POD—beyond which further increases yielded no additional reduction—was 30 IU.

We did not conduct a meta-analysis of hypoglycemia incidence because most included studies reported that both the insulin and placebo groups had no hypoglycemic events, indicating a favourable but not rigorously or systematically assessed safety signal.

### 4.1 Relationship to previous studies

The present study extends previous meta-analyses by examining the dose–response relationship as well as the overall effect of intranasal insulin on POD. Neither Zhang et al.^17^ nor Amin et al.^18^ modelled insulin dose as a continuous exposure, both pooled trials comparing insulin with placebo despite doses ranging from 20 to 80 IU across studies. Our dose–response analysis used restricted cubic splines to characterise how the modelled effect varied with dose, identifying a nadir at 30 IU and a plateau at higher doses. This provides information beyond a pooled pairwise estimate and supports further evaluation of dosing in randomised trials. Our literature search was updated through November 2025, extending beyond the search cut-offs of Zhang et al.^17^ (1 July 2025) and Amin et al.^18^ (December 2024). We also applied the GRADE framework to rate the certainty of evidence with explicit domain-level downgrading, which was not reported by Amin et al.^18^ Finally, we quantified between-study heterogeneity in the dose–response model using the VPC, which was not reported in the previous meta-analyses.

Multiple mechanisms may underlie intranasal insulin’s reduction in the incidence of POD. One possible mechanism is that insulin elevates cerebral glucose concentration, as shown by Yang Mi et al.,^32^ who found that the insulin group had significantly higher central glucose concentration than the placebo group.

Also, POD could be partially affected by the brain developing insulin resistance (as in patients with type 2 diabetes), characterised by reduced neural sensitivity and responsiveness to insulin, leading to reduced insulin uptake.^33^ Intranasal insulin can counteract this mechanism by directly increasing brain insulin levels.

Another possible indirect mechanism by which intranasal insulin can decrease POD is increased central and peripheral osteocalcin levels, as shown by Yang Mi et al.^32^

Although the evidence for osteocalcin’s positive cognitive effects is limited, primarily because it has been tested in animal studies, it remains one of the potential mechanisms by which intranasal insulin can improve cognitive function and decrease the incidence of delirium.

Huang et al.,^25^ in an RCT involving patients undergoing radical resection of oesophageal cancer, also found that the insulin group administered 30 IU had a lower incidence of POD than the group given 20 IU, supporting the superiority of 30 IU. However, another RCT involving patients undergoing hip arthroplasty for hip fracture, Li et al.^27^ found that although both 20 IU and 40 IU doses of insulin decreased POD compared to placebo, there was no significant difference in POD between the two doses when compared directly.

Combining these findings suggests that 20 IU may be the minimum dose required to reduce POD, and increasing the dose to 30 IU could provide maximum neuroprotection without causing serious side effects. Higher insulin doses, such as 160 IU and 240 IU, were not represented in the dose–response curve because they were reported in a single nonrandomised trial by Nakadate et al.,^34^ with a small sample size, making it difficult to draw definitive conclusions regarding these doses.

### 4.2 Recommendations for future research

We recommend that future research conduct more multicentre RCTs with larger sample sizes to explore different intranasal insulin doses. It is particularly important to investigate whether increasing the insulin dose is associated with higher rates of adverse events. Additionally, future trials should standardise insulin treatment regimens and the assessment of POD. Further research should also examine whether the effect of intranasal insulin varies by surgical procedure, as definitive conclusions about its efficacy across procedures are essential. Differences in efficacy across surgical procedures are expected because the magnitude of the systemic and neuroinflammatory response—the presumed mechanistic driver of POD—varies substantially by procedure (e.g., cardiopulmonary bypass in cardiac surgery versus orthopaedic or gastrointestinal surgery). Consistent with this, our exploratory dose–response subgroup analysis suggested a lower nadir dose for orthopaedic surgery (22 IU) than for cardiac surgery (29 IU) (Fig. 6A); although the CIs overlapped, this difference should be considered hypothesis-generating rather than definitive. Future adequately powered trials in specific surgical populations are needed to determine whether procedure-specific dosing is warranted.

### 4.3 Strengths and limitations

Our study has several strengths. First, we used a robust methodological approach, dose–response meta-analysis, that included multiple insulin doses. This not only provided an overall effect of insulin but also identified the optimal dose for reducing POD. All included studies were RCTs. We also utilised the GRADE framework to assess the certainty of evidence.

However, limitations include the small number of studies (only 892 patients). All but one of the included trials were conducted in China; the resulting geographic and population homogeneity limits the generalisability of our findings to other regions and healthcare systems until confirmed in more diverse cohorts. Additionally, some of the included studies used different insulin regimens: some administered insulin only before anaesthesia, while others continued it postoperatively. Furthermore, the studies included different surgical populations (cardiac, orthopaedic, gastrointestinal) and different types of anaesthesia (general and regional), and despite conducting a subgroup analysis to identify the effect of intranasal insulin in each surgical population, the evidence for these groups was not definitive due to the limited sample size for each subgroup. Included trials also differed in the timing and frequency of insulin administration, ranging from a single intraoperative dose to repeated dosing over several preoperative and postoperative days; because only one trial used a single-dose regimen, we could not formally test dosing schedule as an effect modifier, and this remains an important variable for future trials to standardise and report. Although all included trials used a validated CAM-based instrument, the specific tool, assessment frequency, and follow-up window for POD ascertainment varied across studies, which may contribute to between-study variability in absolute POD incidence. Because only eight trials were included, most of which were single-centre in design, our findings may be susceptible to institutional practice effects such as local anaesthetic protocols or delirium-screening practices. Consequently, future multicentre trials are necessary to establish external validity and generalisability.

## 5 Conclusion

Intranasal insulin probably reduces POD in adult surgical patients (moderate-certainty evidence), with a dose–response analysis suggesting 30 IU may provide the greatest benefit. Reported rates of hypoglycaemia were low across included trials, but no trial was adequately powered to assess safety, and mortality and major complications were not systematically reported; therefore, no firm conclusion regarding overall safety can be drawn from the current evidence. Larger, multicentre trials with prespecified, systematically collected safety outcomes are needed to confirm both the optimal regimen and the safety profile of intranasal insulin.

## CRediT authorship contribution statement

Omar Saeed, Abdelrahman Saeed, and Ali M. Abdelaziz contributed to the study’s conceptualisation and protocol development. Omar Saeed, Abdelrahman Saeed, and Ali M. Abdelaziz performed study screening, data extraction, and risk-of-bias assessment. Mohamedhen Vall Nounou, Sung Ryul Shim, and M.E. conducted the statistical analyses and interpreted the data. Omar Saeed, Abdelrahman Saeed, Ali M. Abdelaziz, Bram Rochwerg, Gerald L. Weinhouse, and Muhammed Elhadi drafted the initial manuscript. Bram Rochwerg, Sung Ryul Shim, Gerald L. Weinhouse, and Muhammed Elhadi provided methodological supervision and critically revised the manuscript. All authors reviewed, edited, and approved the final version of the manuscript.

## Data availability statement

All data analysed in this study were derived from peer-reviewed published articles and publicly available sources. The datasets supporting the conclusions of this article are available from the corresponding author upon reasonable request and can be independently obtained from the original publications.

## Consent for publication

Not applicable. This manuscript does not contain any individual patient data, images, or identifiable information requiring consent for publication.

## Declaration of AI usage

In the preparation of this systematic review and meta-analysis, artificial intelligence tools were used solely for grammar correction and style enhancement. All scientific content, data interpretation, and intellectual analyses were conducted exclusively by the authors. The use of artificial intelligence was limited to improving the clarity, readability, and linguistic accuracy of the manuscript without altering the scientific meaning or findings.

## Funding

This research did not receive any specific grant from funding agencies in the public, commercial, or not-for-profit sectors.

## Conflict of interest

The authors declare that they have no competing interests.
