# Supplementary material for: Effect of intranasal insulin on postoperative delirium: A systematic review and dose–response meta-analysis
Source: Crit Care Resusc. 2026 Oct 27;28(4):100227. doi: 10.1016/j.ccrj.2026.100227 (PMC13734877; doi:10.1016/j.ccrj.2026.100227)
Supplement: Supplementary file 1 [file mmc1.docx]

These supplementary materials for the article entitled:

**Effect of Intranasal Insulin on Postoperative Delirium: Systematic Review and Dose–Response Meta-analysis**

Table contents

[**Table S1.** PRISMA checklist table	2](#_Toc240233093)

[**Table S2.** PubMed, Scopus, Web of Science, and Cochrane data search results.	6](#_Toc240233094)

[**Table S3.** Summary characteristics of the included studies	7](#_Toc240233095)

[**Table S4**. Baseline characteristics of the included studies	8](#_Toc240233096)

[**Table S5**. Perioperative intranasal insulin (INI) dosing regimens in included studies	8](#_Toc240233097)

[**Figure S1.** POD (surgery type subgroups)	9](#_Toc240233098)

[**Figure S2.** POD (anesthetic technique subgroups)	9](#_Toc240233099)

[**Figure S3.** POD dose response (linear model)	10](#_Toc240233100)

[**Figure S4.** POD dose response (quadratic model)	11](#_Toc240233101)

[**Figure S5.** POD dose-response, adjusted for age, sex, and BMI (restricted cubic spline model)	12](#_Toc240233102)

[**Figure S6.** Variance partition coefficient (VPC) curve, adjusted for age, sex, and BMI	13](#_Toc240233103)

[**Table S6.** The GRADE framework table	14](#_Toc240233104)

## **Table S1.** PRISMA checklist table

| **Section and Topic** | **Item #** | **Prisma 2020 Checklist item for abstract** | **Reported (Yes/No)** |
| --- | --- | --- | --- |
| **TITLE** | | |  |
| Title | 1 | Identify the report as a systematic review. | Yes |
| **BACKGROUND** | | |  |
| Objectives | 2 | Provide an explicit statement of the main objective(s) or question(s) the review addresses. | Yes |
| **METHODS** | | |  |
| Eligibility criteria | 3 | Specify the inclusion and exclusion criteria for the review. | Yes |
| Information sources | 4 | Specify the information sources (e.g. databases, registers) used to identify studies and the date when each was last searched. | Yes |
| Risk of bias | 5 | Specify the methods used to assess risk of bias in the included studies. | Yes |
| Synthesis of results | 6 | Specify the methods used to present and synthesise results. | Yes |
| **RESULTS** | | |  |
| Included studies | 7 | Give the total number of included studies and participants and summarise relevant characteristics of studies. | Yes |
| Synthesis of results | 8 | Present results for main outcomes, preferably indicating the number of included studies and participants for each. If meta-analysis was done, report the summary estimate and confidence/credible interval. If comparing groups, indicate the direction of the effect (i.e. which group is favoured). | Yes |
| **DISCUSSION** | | |  |
| Limitations of evidence | 9 | Provide a brief summary of the limitations of the evidence included in the review (e.g. study risk of bias, inconsistency and imprecision). | No |
| Interpretation | 10 | Provide a general interpretation of the results and important implications. | Yes |
| **OTHER** | | |  |
| Funding | 11 | Specify the primary source of funding for the review. | No |
| Registration | 12 | Provide the register name and registration number. | No |

| **Section/Topic** | **Item #** | **Prisma 2020 Checklist item for manuscript** | **Location** |
| --- | --- | --- | --- |
| **TITLE** | | |  |
| Title | 1 | Identify the report as a systematic review. | 1 |
| **ABSTRACT** | | |  |
| Abstract | 2 | See the PRISMA 2020 for Abstracts checklist. |  |
| **INTRODUCTION** | | |  |
| Rationale | 3 | Describe the rationale for the review in the context of existing knowledge. | 4-6 |
| Objectives | 4 | Provide an explicit statement of the objective(s) or question(s) the review addresses. | 6 |
| **METHODS** | | |  |
| Eligibility criteria | 5 | Specify the inclusion and exclusion criteria for the review and how studies were grouped for the syntheses. | 7-8 |
| Information sources | 6 | Specify all databases, registers, websites, organisations, reference lists and other sources searched or consulted to identify studies. Specify the date when each source was last searched or consulted. | 6 |
| Search strategy | 7 | Present the full search strategies for all databases, registers and websites, including any filters and limits used. | 6 |
| Selection process | 8 | Specify the methods used to decide whether a study met the inclusion criteria of the review, including how many reviewers screened each record and each report retrieved, whether they worked independently, and if applicable, details of automation tools used in the process. | 7 |
| Data collection process | 9 | Specify the methods used to collect data from reports, including how many reviewers collected data from each report, whether they worked independently, any processes for obtaining or confirming data from study investigators, and if applicable, details of automation tools used in the process. | 7 |
| Data items | 10a | List and define all outcomes for which data were sought. Specify whether all results that were compatible with each outcome domain in each study were sought (e.g. for all measures, time points, analyses), and if not, the methods used to decide which results to collect. | 7 |
|  | 10b | List and define all other variables for which data were sought (e.g. participant and intervention characteristics, funding sources). Describe any assumptions made about any missing or unclear information. | 7 |
| Study risk of bias assessment | 11 | Specify the methods used to assess risk of bias in the included studies, including details of the tool(s) used, how many reviewers assessed each study and whether they worked independently, and if applicable, details of automation tools used in the process. | 7 |
| Effect measures | 12 | Specify for each outcome the effect measure(s) (e.g. risk ratio, mean difference) used in the synthesis or presentation of results. | 8 |
| Synthesis methods | 13a | Describe the processes used to decide which studies were eligible for each synthesis (e.g. tabulating the study intervention characteristics and comparing against the planned groups for each synthesis (item #5)). | 8 |
|  | 13b | Describe any methods required to prepare the data for presentation or synthesis, such as handling of missing summary statistics, or data conversions. | Not Reported |
|  | 13c | Describe any methods used to tabulate or visually display results of individual studies and syntheses. | Not Reported |
|  | 13d | Describe any methods used to synthesize results and provide a rationale for the choice(s). If meta-analysis was performed, describe the model(s), method(s) to identify the presence and extent of statistical heterogeneity, and software package(s) used. | 8 |
|  | 13e | Describe any methods used to explore possible causes of heterogeneity among study results (e.g. subgroup analysis, meta-regression). | 8 |
|  | 13f | Describe any sensitivity analyses conducted to assess robustness of the synthesized results. | 8 |
| Reporting bias assessment | 14 | Describe any methods used to assess risk of bias due to missing results in a synthesis (arising from reporting biases). | 7 |
| Assessment | 15 | Describe any Certainty methods used to assess certainty (or confidence) in the body of evidence for an outcome. | 8 |
| **RESULTS** | | |  |
| Study selection | 16a | Describe the results of the search and selection process, from the number of records identified in the search to the number of studies included in the review, ideally using a flow diagram. | 9 |
|  | 16b | Cite studies that might appear to meet the inclusion criteria, but which were excluded, and explain why they were excluded. | Not Reported |
| Study characteristics | 17 | Cite each included study and present its characteristics. | 9 |
| Risk of bias in studies | 18 | Present assessments of risk of bias for each included study. | 10 |
| Results of individual studies | 19 | For all outcomes, present, for each study: (a) summary statistics for each group (where appropriate) and (b) an effect estimate and its precision (e.g. confidence/credible interval), ideally using structured tables or plots. | 9-10 |
| Results of syntheses | 20a | For each synthesis, briefly summarise the characteristics and risk of bias among contributing studies. | 9-10 |
|  | 20b | Present results of all statistical syntheses conducted. If meta-analysis was done, present for each the summary estimate and its precision (e.g. confidence/credible interval) and measures of statistical heterogeneity. If comparing groups, describe the direction of the effect. | 10-11, 14 |
|  | 20c | Present results of all investigations of possible causes of heterogeneity among study results. | 10-11 |
|  | 20d | Present results of all sensitivity analyses conducted to assess the robustness of the synthesized results. | 10 |
| Reporting biases | 21 | Present assessments of risk of bias due to missing results (arising from reporting biases) for each synthesis assessed. | Not Reported |
| Certainty of evidence | 22 | Present assessments of certainty (or confidence) in the body of evidence for each outcome assessed. | 10 |
| **DISCUSSION** | | |  |
| Discussion | 23a | Provide a general interpretation of the results in the context of other evidence. | 11-13 |
|  | 23b | Discuss any limitations of the evidence included in the review. | 13-14 |
|  | 23c | Discuss any limitations of the review processes used. | 13-14 |
|  | 23d | Discuss implications of the results for practice, policy, and future research. | 13-14 |
| **OTHER INFORMATION** | | |  |
| Registration and protocol | 24a | Provide registration information for the review, including register name and registration number, or state that the review was not registered. | 6 |
|  | 24b | Indicate where the review protocol can be accessed, or state that a protocol was not prepared. | 6 |
|  | 24c | Describe and explain any amendments to information provided at registration or in the protocol. | Not Reported |
| Support | 25 | Describe sources of financial or non-financial support for the review, and the role of the funders or sponsors in the review. | 16 |
| Competing interests | 26 | Declare any competing interests of review authors. | 15 |
| Availability of data, code and other materials | 27 | Report which of the following are publicly available and where they can be found: template data collection forms; data extracted from included studies; data used for all analyses; analytic code; any other materials used in the review. | 15 |

## **Table S2.** PubMed, Scopus, Web of Science, and Cochrane data search results.

Date of search: December 2024 and updated in November 2025

| **Data Base** | **Search Query** | **N** |
| --- | --- | --- |
| **PubMed**  **All fields** | ("Intranasal Insulin" or “intranasal” or "Nasal Insulin " or "insulin spray") and ("Postoperative Delirium" or "POD" or Delirium or Disorientation or confusion or "Acute Confusional State") | **145** |
| **Search update (November 2025)** |  | **27** |
| **Scopus**  TITLE-ABS-KEY | ("Intranasal Insulin" or “intranasal” or "Nasal Insulin " or "insulin spray") and ("Postoperative Delirium" or "POD" or Delirium or Disorientation or confusion or "Acute Confusional State") | **229** |
| **Search update (November 2025)** |  | **37** |
| **Web Of Science**  All fields | ("Intranasal Insulin" or “intranasal” or "Nasal Insulin " or "insulin spray") and ("Postoperative Delirium" or "POD" or Delirium or Disorientation or confusion or "Acute Confusional State") | **188** |
| **Search update (November 2025)** |  | **33** |
| **Cochrane**  All text | ("Intranasal Insulin" or “intranasal” or "Nasal Insulin " or "insulin spray") and ("Postoperative Delirium" or "POD" or Delirium or Disorientation or confusion or "Acute Confusional State") | **213** |
| **Search update (November 2025)** |  | **4** |

## **Table S3.** Summary characteristics of the included studies

| **Study ID** | **Study design** | **Country** | **Sample size** | **Study duration** | **Dose of insulin** | **Type of surgery** | **Inclusion criteria** | **Conclusion** |
| --- | --- | --- | --- | --- | --- | --- | --- | --- |
|  |  |  |  |  |  |  |  |  |
| Huang 2023 | RCT | China | 90 | Between January 2022 and May 2022 | 20IU,30IU | Radical resection for esophageal cancer with a combined thoracoscopy and laparoscopy under general anesthesia | age ≥65 years, American Society of Anesthesiologists (ASA) physical status I to III, body mass index (BMI) ≤28 kg/m²) | Our trial results suggest that in elderly patients who undergo radical resection for esophageal cancer under combined thoracoscopy and laparoscopy, repeated intranasal injections of 30 U insulin may significantly reduce the prevalence of POD and the expression of τ and Aβ proteins at 3 days postoperatively. |
| Huang 2024 | RCT | China | 71 | Between August 2021 and January 2022 | 20IU | Heart valve replacement surgery with a procedure that took 2–6 hours under cardiopulmonary bypass (CPB) | Patients with an American Society of Anesthesiologists (ASA) physical status of II or III, good Mini-Mental State Examination (MMSE) | The results indicate that repeated nasal insulin administration before surgery may reduce the incidence of POD within 3 days after surgery, improve preoperative sleep quality, and reduce preoperative serum cortisol concentration in patients with rheumatic heart disease undergoing valve replacement surgery. |
| Yang Mi 2025 | RCT | China | 195 | Between 1 December 2022 and 18 July 2023 | 40IU | Elective joint replacement | aged 65 years and older, (ASA) physical status I-III, elective joint replacement surgery under combined epidural anesthesia, and stayed in the hospital after surgery for more than 5 days. | Intranasal insulin notably reduced the incidence and severity of POD in elderly patients undergoing joint replacement and significantly improved central and peripheral osteocalcin levels and peripheral insulin sensitivity. |
| Roque 2021 | RCT | Canada | 115 | Between September 2016 and March 2018 | 40IU/80IU | Cardiopulmonary Bypass (CPB) | patients scheduled for elective cardiac surgery for 18 years and older, and who could give written informed consent | Intranasal insulin administration at doses up to 80 IU did not cause clinically significant hypoglycemia. |
| Sun 2024 | RCT | China | 128 | From July 2021 to  March 2022 | 40IU | Spinal surgery/ Lower extremity surgery/Pancreatic surgery | age ≥65 years; (2) scheduled to undergo elective pancreatic surgery or orthopedic surgery with general anesthesia; (3) American Society of Anesthesiologists (ASA) physical status score of Ⅱ−Ⅲ; and (4) expected operation time of ≥2 hours | Administration of intranasal insulin decreased the incidence of POD and inhibited postoperative inflammation in older patients undergoing elective pancreatic surgery or orthopaedic surgery with general anaesthesia. |
| Li 2025 | RCT | China | 130 | Between July 2023 and February 2024 | 20IU, 40IU | Unilateral hip arthroplasty or closed reduction and intramedullary nailing | Patients, aged ≥65 years, with hip fractures who were scheduled for unilateral hip arthroplasty or closed reduction and intramedullary nailing under spinal anesthesia with body mass index (BMI) ≤24 kg/m² and American Society of Anesthesiologists (ASA) class I–III status | Intranasal insulin administration at doses of 20 U and 40 U effectively reduces the incidence of POD in older patients with hip fractures without lowering peripheral blood glucose levels. |
| Yang Ming 2025 | RCT | China | 71 | Between 4 March 2024 and  18 October, 2024 | 20IU | Selective cardiac surgery with cardiopulmonary bypass | Patients aged 45–65 years scheduled for elective cardiac surgery and capable of engaging in concise communication with the investigator, with a Mini-Mental Status Examination (MMSE) score greater than 17 | INI stabilizes postoperative sleep architecture, thereby reducing POD incidence and enhancing cognitive recovery in middle-aged CPB patients. These findings position INI as a targeted intervention for perioperative neurocognitive protection, particularly in populations vulnerable to circadian disruption. |
| Wang 2025 | RCT | China | 92 | between June and December, 2024 | 20IU | Hip fracture surgery | Patients aged ≥ 60 years, body mass index (BMI): 18–28 kg/m2, scheduled for hip fracture surgery under GA, American Society of Anesthesiologists (ASA) classification of physical status I–III | Perioperative INI administration reduced the incidence of POD in elderly patients undergoing hip fracture surgery with GA. |

## **Table S4**. Baseline characteristics of the included studies

| **Study ID** | **Groups** | **Age (Years)  Mean (SD)** | **Sex (Male)  n (%)** | **BMI Mean (SD)** | **LVEF (%) Mean (SD)** | **Hct (%) Mean (SD)** | **ASA classification** | | |  |  |  |  |  |  |
| --- | --- | --- | --- | --- | --- | --- | --- | --- | --- | --- | --- | --- | --- | --- | --- |
|  |  |  |  |  |  |  | **Grade I** | **Grade II** | **Grade III** | **Hypertension** | **Smoking** | **COPD** | **Atrial fibrillation** | **Diabetes mellitus** | **Alcohol use** |
| Huang 2023 | control (saline) | 68.5(2.34) | 16(53.3%) | 23.56(2.69) |  |  | 11 (36.7%) | 16 (53.3%) | 3 (10.0%) |  |  |  |  |  |  |
|  | insulin20U | 68.08(3.31) | 20(66.7%) | 22.40(2.09) |  |  | 10 (33.3%) | 19 (63.3%) | 1 (3.3%) |  |  |  |  |  |  |
|  | insulin30U | 68.3(3.89) | 14 (46.7%) | 22.82(2.23) |  |  | 12 (40.0%) | 17 (56.7%) | 1 (3.3%) |  |  |  |  |  |  |
| Huang 2024 | control (saline) | 55(10) | 14(40%) | 22.34 (2.91) |  |  |  | 9 (25.7%) | 26 (74.3%) |  |  |  |  |  |  |
|  | insulin 20U | 53(12) | 12 (33.3%) | 23.25 (2.51) |  |  |  | 6 (16.7%) | 30 (83.3%) |  |  |  |  |  |  |
| Yang Mi 2025 | Saline | 73(6.68) | 26(26.26) | 23.84(3.72) |  |  |  |  |  | 42(42.42) | 8(8.08%) |  |  | 22(22.22) | 4(4.04) |
|  | Insulin 40 | 72.8(6.0) | 24(25) | 23.06(3.09) |  |  |  |  |  | 56(58.33) | 12(12.5) |  |  | 24(25) | 4(4.17) |
| Roque 2021 | Saline (non-T2DM) | 70 (9) | 22 (91.66%) | 27 (5) | 57.00(14.97) | 40 (4) |  |  |  | 20 (83%) | 5 (20,8%) | 3 (12%) | 1 (4%) |  |  |
|  | Insulin40IU | 68 (8) | 19 (79.16%) | 27 (5) | 60.00(7.88) | 42 (3) |  |  |  | 21 (88%) | 3 (12%) | 5 (20,8%) | 1 (4%) |  |  |
|  | Insulin80IU | 66 (12) | 17 (70.83%) | 27 (6) | 58.33(11.82) | 42 (4) |  |  |  | 19 (79%) | 5 (20%) | 3 (12%) | 2 (8%) |  |  |
|  | Saline(T2DM) | 68 (8) | 8 (61.53%) | 29 (5) | 45(29.07) | 41 (5) |  |  |  | 11 (84.6%) | 1 (8%) | 1 (8%) | 2 (15%) | 13 (100%) |  |
|  | Insulin40IU | 67 (8) | 12 (80%) | 32 (7) | 46.67(24.54) | 40 (4) |  |  |  | 14 (93%) | 2 (13%) | 5 (33%) | 1 (7%) | 15 (100%) |  |
|  | Insulin80IU | 66 (10) | 10 (66.66%) | 29 (4) | 50.00(16.36) | 41 (5) |  |  |  | 15 (100%) | 1 (7%) | 5 (33%) | 1 (7%) | 15 (100%) |  |
| Sun 2024 | Saline | 68.86(3.63) | 26(40.6) | 25.17 (3.65) |  |  |  | 59(92.2%) |  | 36(56.2%) | 6(9.4%) |  |  | 22(34.4%) | 7(10.9%) |
|  | Insulin40IU | 68.69(3.17) | 22(34.4) | 26.19 (3.59) |  |  |  | 57(89.1%) |  | 35(54.7%) | 8(12.5%) |  |  | 29(45.3%) | 8(12.5%) |
| Li 2025 | placebo (saline) | 81.25 (2.97) | 12 (27.9) | 20.7 (1.9) |  |  |  | 6 (14%) | 37 (86%) |  |  |  |  | 10 (23.3%) |  |
|  | insulin 20 U | 79 (2.72) | 16 (35.6) | 20.7 (1.9) |  |  |  | 6 (13%) | 39 (87%) |  |  |  |  | 11 (24.4%) |  |
|  | insulin 40U | 81 (3.21) | 9 (21.4) | 20.7 (2) |  |  |  | 5 (12%) | 37 (88%) |  |  |  |  | 12 (28.6%) |  |
| Yang Ming 2025 | Saline | 55.92 (4.78) | 13 (36.11) | 23.71 (2.97) |  |  |  |  | 36 (100%) | 5 (13.89 %) | 9 (25 %) |  | 14 (38.9 %) |  | 9 (25 %) |
|  | Insulin 20 | 55.86 (5.65) | 16 (45.71) | 25.37 (2.01) |  |  |  |  | 35 (100%) | 4 (11.43 %) | 11 (31.4 %) |  | 12 (34.2 %) |  | 6 (17.1 %) |
| Wang 2025 | Saline | 71.8 (6.87) | 11 (23.9) | 23.41 (3.02) |  |  |  | 37 (80.4%) | 9 (19.6%) | 29 (63%) |  |  |  |  |  |
|  | Insulin 20 | 72.41 (6.6) | 7 (15.2) | 23.1 (2.98) |  |  |  | 39 (84.8%) | 7 (15.2%) | 25 (54.3%) |  |  |  |  |  |

## **Table S5**. Perioperative intranasal insulin (INI) dosing regimens in included studies

| **Study** | **Preoperative Dosing** | **Intraoperative Dosing** | **Postoperative Dosing** | **Total Doses** |
| --- | --- | --- | --- | --- |
| **Huang 2023** | 2 days before surgery: BID (09:00 & 19:00). Final dose: 10 min before induction on Day 0 (morning). Doses: Day -2 (2), Day -1 (2), Day 0 (1) = 5 | None | None | 5 |
| **Huang 2024** | 2 days before surgery: BID (09:00 & 19:00). Final dose: 10 min before induction on Day 0 (morning). Doses: Day -2 (2), Day -1 (2), Day 0 (1) = 5 | None | None | 5 |
| **Li 2025** | Day -1: 19:00 (1 dose). Day 0: 50 min before induction (1 dose). Total preop = 2 | None | Day 0: 19:00 (1 dose). Total postop = 1 | 3 |
| **Yang Mi 2025** | 3 days before surgery: BID (Days -3, -2, -1, and Day 0 morning). Preop doses = 7 (Days -3 to -1: 6 + Day 0 AM: 1) | None | From Day 0 evening through Day 5: BID. Doses: Day 0 PM, Days 1-5 (10 doses) = 11 | 18 |
| **Roque 2021** | None | Single dose given after induction of general anesthesia (in OR) | None | 1 |
| **Sun 2024** | Day 0: 5 min before induction (1 dose) | None | Days 1, 2, 3: once daily (1 dose each) | 4 |
| **Wang 2025** | 2 days before surgery: BID (09:00 & 19:00). Covers Days -2, -1, and Day 0 morning. Preop doses = 5 | None | Day 0 evening + Days 1 and 2: BID (09:00 & 19:00). Postop doses = 5 | 10 |
| **Yang Ming 2025** | Day 0: 1 hour before induction (1 dose) | None | Days 1 and 2: once daily (08:00). Postop doses = 2 | 3 |

## **Figure S1.** POD (surgery type subgroups)

## **Figure S2.** POD (anesthetic technique subgroups)

## **Figure S3.** POD dose response (linear model)

## **Figure S4.** POD dose response (quadratic model)

## **Figure S5.** POD dose-response, adjusted for age, sex, and BMI (restricted cubic spline model)

## **Figure S6.** Variance partition coefficient (VPC) curve, adjusted for age, sex, and BMI

## **Table S6.** The GRADE framework table

| Outcome | Number of Studies (Participants) | Risk of Bias | Inconsistency (I²) | Indirectness | Imprecision | Publication bias | Other considerations | Overall Quality |
| --- | --- | --- | --- | --- | --- | --- | --- | --- |
| Postoperative delirium (POD) | 8 studies (891) | No downgrading | No downgrading | No downgrading | Downgraded | No downgrading | None | ⊕⊕⊕◯ MODERATE |

Explanations

(1) Risk of bias downgrades were applied if ≥ 50% of studies contributing to the analysis had a high risk of bias or some concerns

(2) Inconsistency downgrades were applied for I² ≥ 50% or the VPC shows moderate to high heterogeneity without adequate explanation

(3) Imprecision downgrades were applied for confidence intervals that included both clinically important benefits and harms or crossed the null, or if the sample size was small and underpowered

(4) Indirectness was assessed based on population, intervention, or outcome differences

(5) Publication bias: Downgraded if the p-value for the funnel plot was less than 0.10. Publication bias was not applicable as the meta-analysis included fewer than 10 studies
